# Supplementary material for: Proposed Mobility Assessments with Simultaneous Full-Body Inertial Measurement Units and Optical Motion Capture in Healthy Adults and Neurological Patients for Future Validation Studies: Study Protocol
Source: Sensors (Basel). 2021 Aug 30;21(17):5833. doi: 10.3390/s21175833 (PMC8434336; doi:10.3390/s21175833)
Supplement: Supplementary file 1 [file sensors-21-05833-s001.zip › sensors-1338135-supplementary.pdf]

Supplementary material

Supplementary Table 1. Information about the placement of the reflective markers and the corresponding name in the data files. See also supplementary figure 1.

Abbreviation used in the data files	Location	Exact description of the location
**Head**		
l/rf_hd	front of the head	Located approximately over the temple
l/rb_hd	back of the head	Placed on the back of the head, roughly in a horizontal plane of the front head markers
**Torso**		
m_ster1	sternum	Placement mid of sternum, just caudal of SC joints
m_ster2	sternum	Caudal and to the left of ster1
m_ster3	sternum	Caudal and to the right of ster1
**Arm**		
l/r_sho	shoulder	Placed on the acromio-clavicular joint
l/r_ua	upper arm	Placed between elbow and shoulder (left side closer to elbow, right side closer to shoulder)
l/r_elbl	elbow	Placed on lateral epicondyle approximating elbow joint axis
l/r_st_hem	humerus epicondylus medialis	Placed on top of humerus epicondylus medialis
l/r_frm	forearm	Placed between wrist and elbow (left side closer to wrist, right side closer to elbow)
l/r_wrr	wrist, radial side	On IMU above the radial styloid
l/r_wru	wrist, ulnar side	On IMU above the ulnar styloid
l/r_hand	dorsal side of hand	Dorsum of the hand just proximal of the head of the second metacarpal
**Pelvis**		
l/r_asis	anterior superior iliac spine	Placed directly on top of anterior superior iliac spine
l/r_psis	posterior superior iliac spine	Placed directly on top of posterior superior iliac spine
**Leg**		
l/r_th	thigh	Marker cluster on lateral side of thigh, contains four markers
l/r_st_fem	femur epicondylus medialis	Placed on the medial epicondyle of the knee
l/r_st_fel	femur epicondylus lateralis	Placed on the lateral epicondyle of the knee
l/r_sk	shank	Marker cluster on lateral side of shank, contains 4 markers

l/r_st_mm	malleolus medialis	Placed on the malleolus medialis
l/r_ank	ankle	Placed on the malleolus lateralis
l/r_heel	heel	Placed on the calcaneous
l/r_toe	toe	Placed on the shoe around the 2nd middle phalanx

IMU = inertial measurement unit; l/r = marker is placed both on the left and right side; m = marker is placed around the midline of the body; st = indicates that this is a static marker that is only on the body during the static calibration trials.

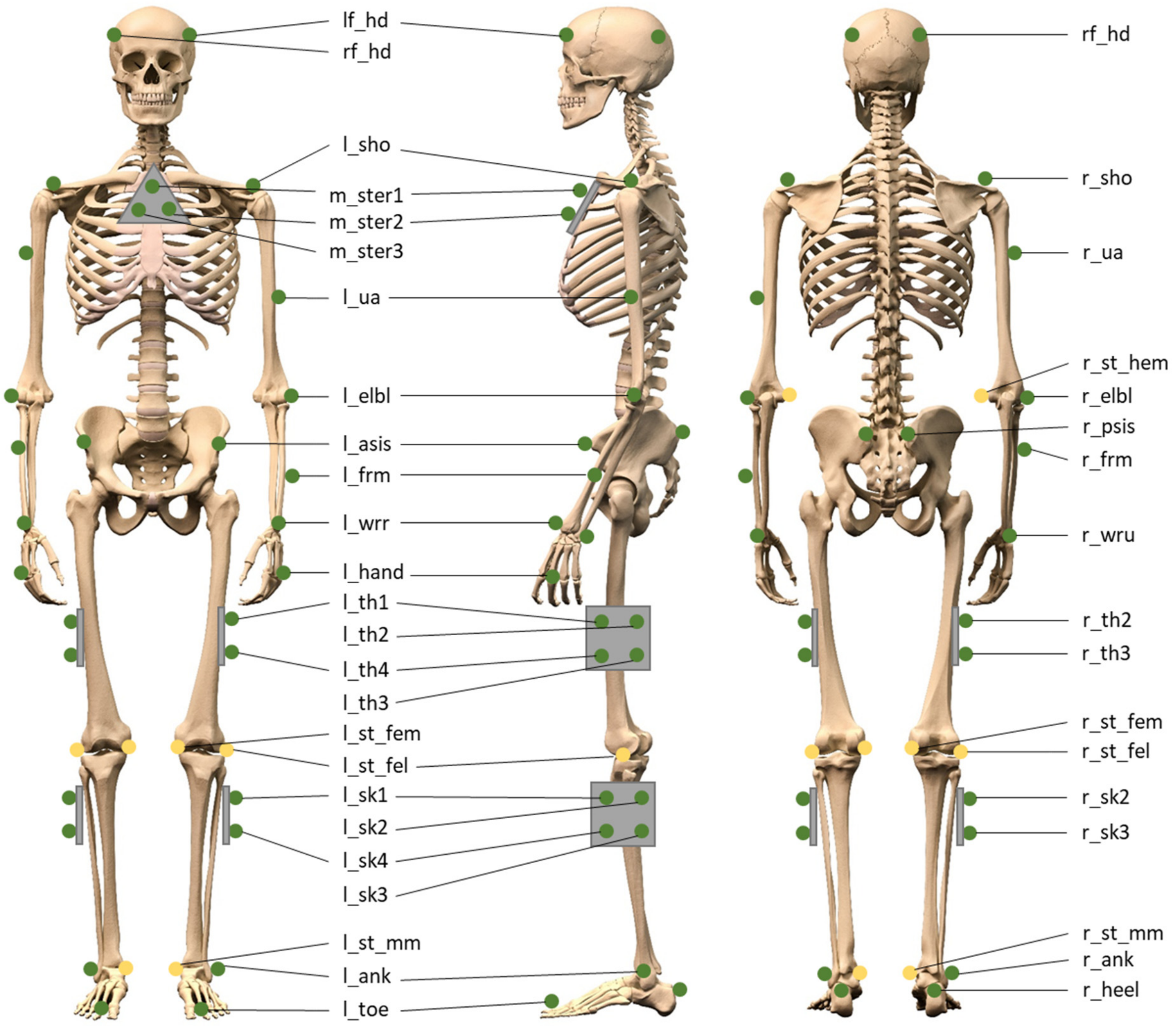

Supplementary Figure 1. Placement of the reflective markers and the corresponding name in the data files. Full names and descriptions can be found in supplementary table 1.

Treadmill split-belt protocol

A subset of the healthy young adults will be asked to participate in two split-belt trials. The speed of the treadmill will be set to the comfortable walking speed from the first part of the first treadmill trial

at which the participant will walk for 60 s. Then the speed of one of the two belts will be reduced with 25% for 120 s. This will be followed by another 60 s with both belts at the same comfortable walking speed. This trial will then be repeated with a speed reduction of the other belt. The order in which belt the speed was reduced first will be randomized.

Choreography assessment

The choreography assessment will exist of a series of movements related to the flexibility of the lower back. The participants will first perform a full flexion and extension of the back. Second, a right and left rotation of the back will be performed, which will be followed by a right and left lateral flexion of the back. Thereafter, the participants will pretend to lift an object from the floor (which is located in front and slightly to the right of the participant), they will lift this above the head and put it back on the floor (in front and slightly to the left of them). This is a movement that combines flexion and rotation of the back. Lastly, the participants will sit down and lift up the right leg while keeping it bended and pretend to put on a sock, which will then be repeated with the left leg.

Supplementary Table 2. Filenames of the IMU and optical motion capture data.

Assessment	Filename (preceded by "omc_" for the optical motion capture data and "imu_" for the IMU data)
Calibration	calibration_#
MDS-UPDRS III	updrs3_#
Treadmill walking tied belt	treadmill
Treadmill walking split-belt	splitbelt_left/splitbelt_right
SPPB – side by side stance	balance_sbs
SPPB – semi-tandem stance	balance_semi
SPPB – tandem stance	balance_tandem
SPPB – first 4 m gait	gait1
SPPB – second 4 m gait	gait2
SPPB – 5 x chair rise	chairrise_fast
Timed up and go	tug
Five time sit to stand test	chairrise_preferred
Choreography	choreo
Straight walking – slow	walk_slow
Straight walking – preferred	walk_preferred
Straight walking – fast	walk_fast
Sideways walking	sideways
Backwards walking	backwards
Obstacle – low	obstacle_low
Obstacle – high	obstacle_high
Slalom	slalom
Single-task – simple reaction time task	reaction
Single-task – numerical stroop task	stroop
Single-task – walking up and down	walk_turn

Dual-task – simple reaction time task and walking	walk_reaction
Dual-task – numerical stroop task and walking	walk_stroop
non-standardized activities of daily living	home_part#

# = a number, sometimes this assessment is performed multiple times or in multiple parts.
